# Spinal Paralysis in the Hyena and Puma

**Published:** 1881-10

**Authors:** Charles A. Todd

**Affiliations:** Medical Director St. Louis Zoological Garden; St. Louis, Mo.


### III.—SPINAL PARALYSIS IN THE HYENA AND PUMA.

Editor of Journal of Comparative Medicine:

At the St. Louis Zoological Garden (Department of the St. Louis Fair Association) there have been several cases, among the Carnivora, of paralysis, dependent upon spinal lesions.

An adult spotted hyena for a number of months, without known cause, had manifested a weakness in the hind parts, as shown by a more or less noticeable "wobbling" of the hind legs in turning. This symptom was followed by decided paralysis, the animal not being able to stand. There were no other symptoms. Upon treatment with strych. sulph., this condition improved and the beast seemed on the road to recovery, though a return to complete health did not seem probable. While passing through a doorway she struck her back, or rather scraped it lightly, and instantly collapsed, falling as though struck down. From this time on the paralysis persisted—her condition gradually weakening. A benevolent individual kindly suggested to blister the back, but did not accept the invitation to walk in and apply his remedy. All attempts at introduction of anything into the cage was greeted by the usual snap, and a hyena's jaws have a phenomenal grip. Death was ushered in by a free bleeding at the mouth and convulsions. At the post-mortem the blood was found to come from the lungs, which were partly engorged. The spleen also was the seat of hæmorrhagic lesion (infarction). The spinal cord in the lumbar region was inflamed and softened through and through.

Three puma cubs, born upon the grounds, and of the same mother, in two successive years have been victims to the same misfortune, but in their cases the disease was probably due to injury from falls, the animals persisting in climbing about and occasionally getting a bad tumble. I understand that a travelling menagerie has lost several lion cubs from spinal affections, and probably this has been an experience in all collections of caged beasts. Two of the pumas I killed, as they, at the end of several months, had become hopelessly crippled in their hind parts. One of them, under the exhibition of strych. sulph., fairly recovered. It at first suffered from a general paralysis of all the limbs. The appetites of all remained voracious, after their kind, the fore-legs and quarters developed, and their tempers showed no sign of degeneracy, they being the most spiteful of all the Carnivora, and prompt to strike out, even when unable to do more than drag themselves to the bars. This unconquerable viciousness seemed strange, as we have five adult pumas, including two males, in a common cage, who live together in peace, and never offer to strike at lookers-on.

Possibly the experience of others in the care of wild beasts may throw some light upon the nature of this spinal affection—supposing it not due to direct injury. I will add that the hyena, during life, passed for a male, the keeper being so assured through its actions, that he was unwilling to credit the discovery, at the dissection, of ovaries and other female properties. The determination of sex in the spotted hyena is very difficult. The superintendent of one of the most important Zoological Gardens in the country stated last year to me that of the two specimens under his charge he did not know the sex of one or the other.

Yours, very truly,

CHARLES A. TODD, A.M., M.D.,

*Medical Director St. Louis Zoological Garden.*

ST. LOUIS, MO.

